# Supplementary material for: Type I and III interferons synergize with TNF to promote virally-triggered damage to the intestinal epithelium
Source: bioRxiv. 2026 Mar 18:2026.01.10.698817. Originally published 2026 Jan 12. Preprint. [Version 2] doi: 10.64898/2026.01.10.698817 (PMC12871235; doi:10.64898/2026.01.10.698817)
Supplement: Supplement 1 [file media-1.pdf]

Supplementary material for

Type I and III interferons synergize with TNF to promote virally-triggered damage to the intestinal epithelium

Lucie Bernard-Raichon[1,2,3,4]*, Jessica A Neil[1,2,5]*, Kayla Kim[3], Thomas Heaney[1,2], Brittany M Miller[3], Damee Moon[2,6], Ashira Lubkin[2,7,8], Ashley L DuMont[2,9], Victor J Torres[2,9], Jordan Axelrad[10], Yu Matsuzawa-Ishimoto[1,2,11], and Ken Cadwell[1,2,3,12]

* Authors contributed equally to this work

Correspondence to: Ken Cadwell (ken.cadwell@pennmedicine.upenn.edu), Yu Matsuzawa-Ishimoto (matsuzawa.yu@tmd.ac.jp)

Supplementary methods

MNV stock generation

MNV.CR6 stock was prepared as previously described[1]. In brief, supernatant from 293T cells transfected with a plasmid containing the MNV.CR6 genome was used to inoculate to RAW264.7 cells. Infectious virus in the supernatant was concentrated by ultracentrifugation and resuspended in endotoxin-free PBS. Virus titer was determined by plaque assay as previously described with few modifications [2]. Diluted virus was incubated on confluent RAW264.7 cells for 1h followed by overlay with 10g/L methylcellulose media (Sigma-Aldrich). Cells were incubated at 37°C for 72 h and stained with 1g/L crystal violet solution.

Intestinal organoid culture

Murine small intestinal crypts were isolated and cultured as described previously using ENR media composed of DMEM/F-12 (ThermoFisher) in the presence of 100IU Penicillin and 100 µg/ml Streptomycin (Corning), 125 µg/ml Gentamicin (ThermoFisher), 2 mM-Glutamine (Corning), 20 ng/ml mEGF (PeproTech), 100 ng/ml mNoggin (R&D systems), and 500 ng/ml mR-Spondin 1 (R&D systems)[3]. Human organoids were generated during previous studies and frozen[4, 5]. For this study, they were thawed and cultured in Human IntestiCult Organoid Growth Medium (Stemcell Technologies). For viability assays, crypts were embedded at 50 crypts /10 µl Matrigel (Stemcell Technologies) and cultured in triplicate for 3 days in 96-well culture plates. Assays were performed in ENR medium for mouse organoids or in differentiation medium for human organoids. Differentiation medium was composed of DMEM/F-12 containing 100ng/mL rhIGF-1 (Biolegend), 50ng/mL hFGF-basic (PeproTech), 1µg/mL rhR-Spondin 1 (R&D systems), 100ng/mL rmWnt-3A (R&D systems), 100ng/mL rmNoggin (R&D systems), 10mM Nicotinamide (Millipore), 10 nM

[Leu15]- Gastrin 1 human (Sigma-Aldrich), 500nM A 83-01 (TOCRIS), 1X B-27 supplement (ThermoFisher) as in[6, 7].

Quantitative qPCR

RNA was extracted from organoids and intestinal tissue using the RNeasy mini kit (Qiagen). DNA was removed using the RNA-free DNase set (Qiagen). cDNA was synthesized using the High-Capacity cDNA reverse transcription kit (Thermo Fisher) according to the manufacturer's protocol. Quantitative PCR was performed on a Roche 480 II LightCycler using SYBR Green I Master mix (Roche). Gene expression was normalized to either *Gapdh* (organoids) or *18S* (tissue). All primers are listed in Table S2.

Immunoblotting

600 small intestinal organoids were seeded in 24 well plates and cultured for 3 days in ENR before cytokine stimulation with 20 ng/ml mTNFα in the presence of either 100 U/ml IFNβ or 1 ng/ml IFNλ2 for 2-24h. Organoids were incubated in lysis buffer (20 mM Tris-HCl, pH 7.4, 150 mM NaCl, 1% Triton X-100, 10% glycerol) containing Halt protease and phosphatase inhibitor cocktail (Thermo Fisher Scientific) and run on a 4%–12% Bis-Tris Plus Gel (Invitrogen) as before[7]. The following antibodies were used for immunoblotting studies: anti–β-actin (AC-15; Sigma-Aldrich), anti–Phospho-MLKL (Ser345), anti-MLKL (Cell Signaling), anti-Phospho-RIP3 (phospho S232; Abcam), anti-RIP3 (AHP1797; AbD Serotec), anti-caspase-1 (AG-20B-0042-C100, AdipoGen) anti-caspase-3 (9662, Cell Signaling), anti-caspase-8 (ALX-804-447, Enzo) and anti-Atg16L (Sigma-Aldrich). Secondary antibodies IRDye 680RD goat anti–rabbit IgG (925-68071) and IRDye 800CW goat anti–mouse IgG (925-32210) were purchased from LI-COR.

Histology and Immunohistochemistry

Quantification of all microscopy data were performed blind. Intestinal sections were prepared and stained as previously described[7]. Terminal deoxynucleotidyl transferase dUTP nick end labeling (TUNEL) staining was performed using the In situ Cell Death Detection POD kit (Roche Diagnostics) on the Discovery XT according to the manufacturer's protocol. Lysozyme staining was performed using anti-lysozyme (ab108508, Abcam) and DAPI immunostaining. All slides were analyzed using a Zeiss AxioObserver.Z1 with Axiocam 503 Mono operated with Zen Blue software. Images were processed and quantified using QuPath. At least 50 villi or crypts per mouse were quantified. Abnormal Paneth cells were defined as Paneth cells showing disordered, depleted and/or diffuse lysozyme staining as in[7, 8] and adapted from[9].

References

1. Strong DW, Thackray LB, Smith TJ, Virgin HW. Protruding domain of capsid protein is necessary and sufficient to determine murine norovirus replication and pathogenesis in vivo. *J Virol* 2012; **86**(6)**:** 2950-2958.

2. Gonzalez-Hernandez MB, Bragazzi Cunha J, Wobus CE. Plaque assay for murine norovirus. *J Vis Exp* 2012; (66)**:** e4297.

3. Matsuzawa-Ishimoto Y, Shono Y, Gomez LE, Hubbard-Lucey VM, Cammer M, Neil J *et al.* Autophagy protein ATG16L1 prevents necroptosis in the intestinal epithelium. *J Exp Med* 2017; **214**(12)**:** 3687-3705.

4. Matsuzawa-Ishimoto Y, Hine A, Shono Y, Rudensky E, Lazrak A, Yeung F *et al.* An intestinal organoid-based platform that recreates susceptibility to T-cell-mediated tissue injury. *Blood* 2020; **135**(26)**:** 2388-2401.

5. Jang KK, Kaczmarek ME, Dallari S, Chen YH, Tada T, Axelrad J *et al.* Variable susceptibility of intestinal organoid-derived monolayers to SARS-CoV-2 infection. *PLoS Biol* 2022; **20**(3)**:** e3001592.

6. Fujii M, Matano M, Toshimitsu K, Takano A, Mikami Y, Nishikori S *et al.* Human Intestinal Organoids Maintain Self-Renewal Capacity and Cellular Diversity in Niche-Inspired Culture Condition. *Cell Stem Cell* 2018; **23**(6)**:** 787-793 e786.

7. Matsuzawa-Ishimoto Y, Yao X, Koide A, Ueberheide BM, Axelrad JE, Reis BS *et al.* The gammadelta IEL effector API5 masks genetic susceptibility to Paneth cell death. *Nature* 2022; **610**(7932)**:** 547-554.

8. Bernard-Raichon L, Venzon M, Klein J, Axelrad JE, Zhang C, Sullivan AP *et al.* Gut microbiome dysbiosis in antibiotic-treated COVID-19 patients is associated with microbial translocation and bacteremia. *Nat Commun* 2022; **13**(1)**:** 5926.

9. Cadwell K, Patel KK, Maloney NS, Liu TC, Ng AC, Storer CE *et al.* Virus-plus-susceptibility gene interaction determines Crohn's disease gene Atg16L1 phenotypes in intestine. *Cell* 2010; **141**(7)**:** 1135-1145.

Supplementary figures

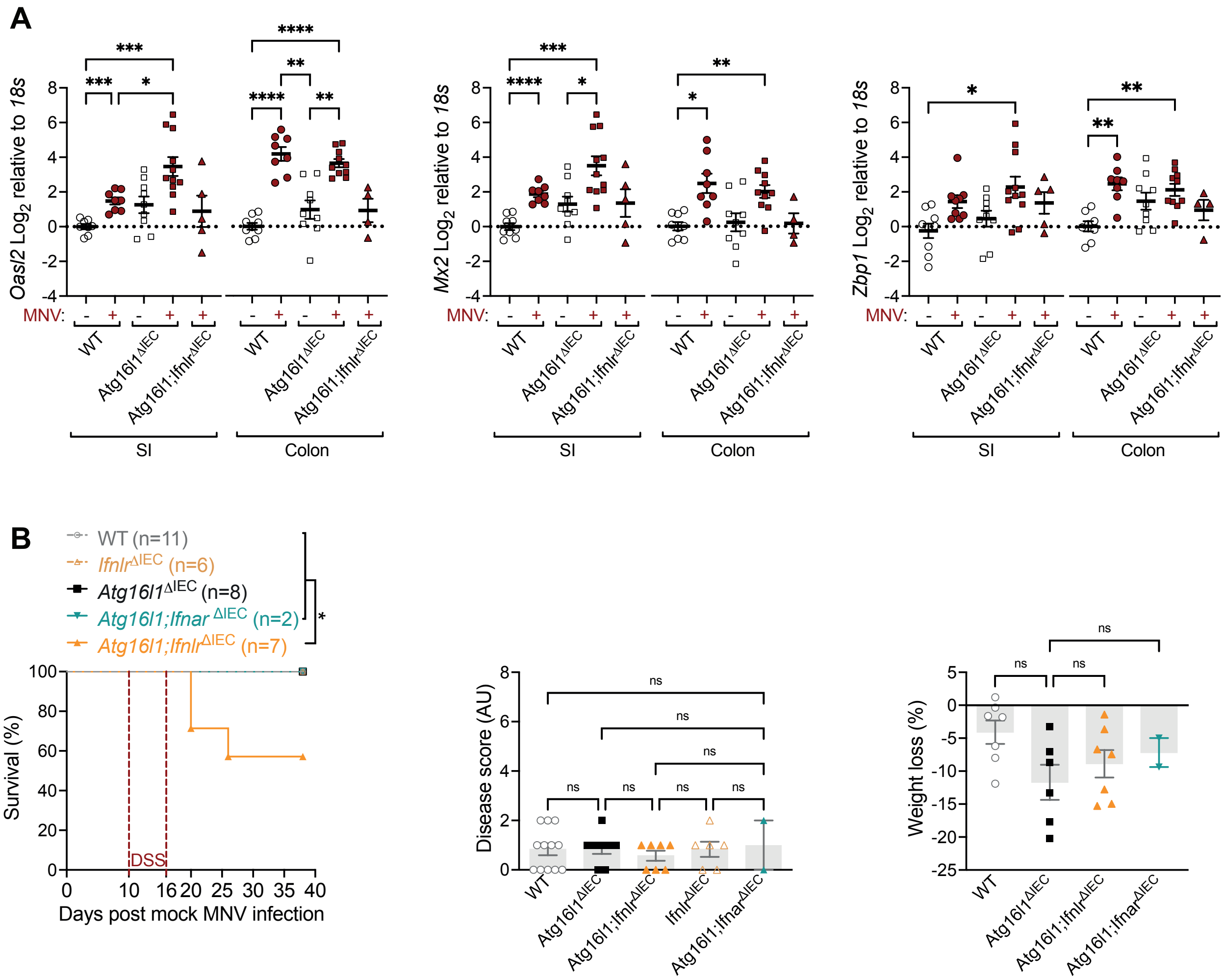

Supplementary figure 1: Characterization of MNV infected and uninfected *Atg16L1*$^{\Delta IEC}$ mice. (A) Normalized RNA levels of *Oasl2*, *Mx2* and *Zbp1* in the ileum (SI) or colon of WT, *Atg16l1*$^{\Delta IEC}$, and *Atg16l1;Ifnlr*$^{\Delta IEC}$ mice infected or not with MNV CR6 for 10 days. **(B)** Survival, clinical score, and weight loss of mock infected WT, *Atg16l1*$^{\Delta IEC}$, *Atg16l1;Ifnlr*$^{\Delta IEC}$ and *Atg16l1;Ifnar*$^{\Delta IEC}$ mice receiving 5% DSS in their drinking water for 6 days. Dots represents individual mice and line (A) or bars (B) represent mean ± SEM from at least two independent experiments analyzed by ordinary one-way ANOVA and Tukey's multiple comparison (area under the curves were compared for B). Related to Fig. 1.

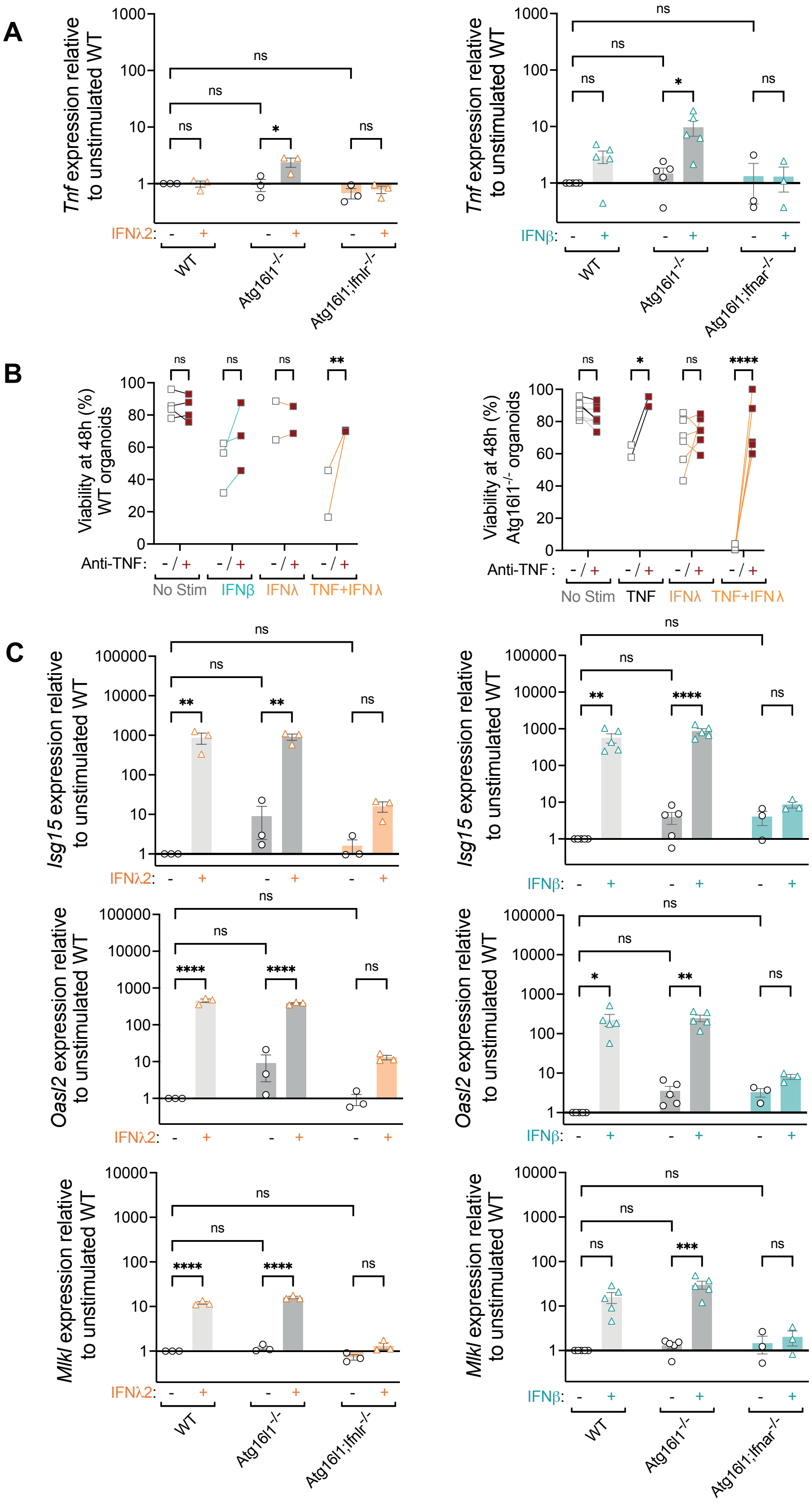
A
B
C
Tnf expression relative to unstimulated WT
IFNλ2:
IFNβ:
WT
Atg16l1-/-
Atg16l1;Ifnlr-/-
Atg16l1;Ifnar-/-
Viability at 48h (%) WT organoids
Viability at 48h (%) Atg16l1-/- organoids
Anti-TNF:
No Stim
IFNβ
IFNλ
TNF+IFNλ
TNF
Isg15 expression relative to unstimulated WT
Oasl2 expression relative to unstimulated WT
Mlkl expression relative to unstimulated WT
ns
*
**
***
****

Supplementary figure 2: Analysis of Tnf, ISG and cell death related transcripts in murine organoids. **(A)** Small intestinal organoids derived from WT, *Atg16l1*$^{\Delta IEC}$, *Atg16l1;Ifnar*$^{\Delta IEC}$, or Atg16l1;Ifnlr$^{\Delta IEC}$ mice were cultivated in ENR medium for 3 days before stimulation with ± 100 U/ml IFNβ or ± 1ng/ml IFNλ2 for 24h. Normalized RNA levels to GAPDH and WT unstimulated organoids are depicted. RNA levels were measured on an average of 600 organoids derived from at least 2 different mice in independent experiments for each condition (as depicted by each symbol). **(B)** Viability of *Atg16l1*$^{+/+}$ (left) or *Atg16l1*$^{-/-}$ (right) organoids treated with 20 µg/ml anti-TNF antibody or isotype control for at least 30min before stimulation with cytokines. **(C)** Normalized RNA levels of ISGs as measured in (A). Bars represent mean ± SEM from at least two independent experiments analyzed by ordinary one-way ANOVA and Tukey's multiple comparison. Relative to Fig. 2 and 3.

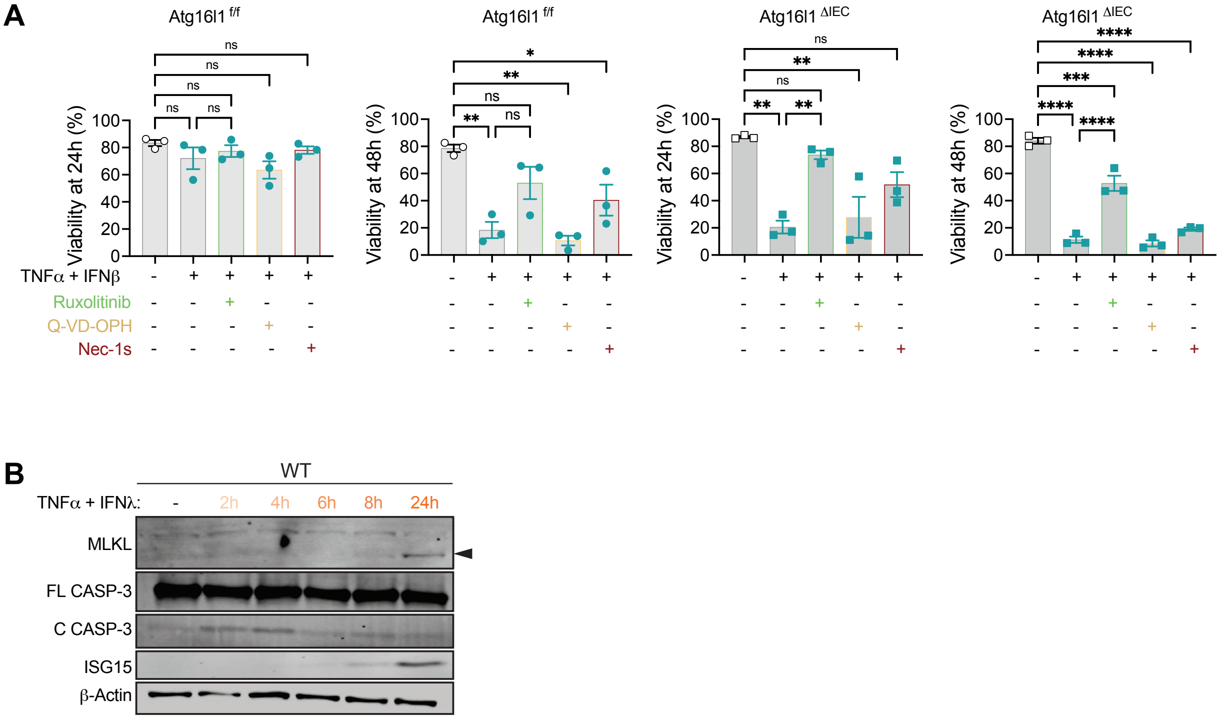

Supplementary figure 3: IFNβ and IFNλ2 contribute to necroptosis and other cell death in small intestinal organoids. Small intestinal organoids were cultivated in ENR medium for 3 days before stimulation with ± 20 ng/ml TNFα ±100U/ml IFNβ,. Viability is determined on the basis of PI staining and morphology before stimulation and every 24h. **(A)** Viability of WT or *Atg16l1*$^{\Delta IEC}$ organoids pre-treated with 100nM Ruxolitinib, 10µM Q-VD-OPh, or 10 µM Nec-1s for at least 30min before TNFα+IFNβ stimulation shown after 24h and 48h. Each symbol represents the average viability observed for each condition on a technical triplicate done in an independent experiment with bars representing mean ± SEM. **(B)** Representative Western blot for analysis of cell death–related proteins. Protein lysate from approximately 600 organoids stimulated for 2-24h with the indicated cytokines was used for each condition. ns = not significant by ordinary one-way ANOVA and Tukey's multiple comparison. Relative to Fig. 3 and 4.

Table S1: Patient information for human specimens used to derive organoids

N°	ATG16L1^{T300A}	Inflammation	Tissue	Sex	Age	Figures	Original study
**1**	(T;T)	non-inflamed	duodenum	F	34	5A-C, E, F	
**2**	(T;A)	non-inflamed	duodenum	M	23	5B, C, E, F	
**3**	(T;A)	non-inflamed	duodenum	M	47	5B, C	
**4**	(T;A)	inflammed	ileum	F	22	5B, C	[4]
**5**	(T;A)	non-inflamed	duodenum	M	37	5B, C	
**6**	(T;A)	non-inflamed	duodenum	F	32	5B, C	
**7**	(T;A)	non-inflamed	duodenum	F	43	5B-E	
**8**	(T;A)	non-inflamed	ascending colon	M	22	5B, C	
**9**	(A;A)	non-inflamed	duodenum	M	21	5A-E	[4]
**10**	(A;A)	non-inflamed	duodenum	M	37	5B, C, E, F	
**11**	(A;A)	non-inflammed	terminal ileum	M	23	5B, C, E, F	[5]

Table S2: characteristic of human serum samples

Patient ID	SARS-CoV-2	BSI micro-organism
A	-	*E. faecium*
B	-	*P. aeruginosa*
C	-	*C. glabrata*
D	-	*C. glabrata*
E	-	*C. glabrata*
F	-	*E. faecalis*
G	-	*E. coli*
H	-	*E. coli*
I	-	*E. cloacae*
J	-	*S. aureus*
K	+	*E. faecium*
L	+	*C. dulinesis, S. aureus*
M	+	*S. aureus*
N	+	*P. aeruginosa*
O	+	*E. cloacae*
P	+	*E. faecalis*
Q	+	*E.coli*
R	+	*Serratia*

Table S3: Primers for RT-PCR

Gene	Forward Primer	Reverse Primer
Gapdh	TGGCCTTCCGTGTTCCTAC	GAGTTGCTGTTGAAGTCGCA
18S	GTAACCCGTTGAACCCCATT	CCATCCAATCGGTAGTAGCG
Mx2	CCAGTTCCTCTCAGTCCCAAGATT	TACTGGATGATCAAGGGAACGTGG
OasL2	GGATGCCTGGGAGAGAATCG	TCGCCTGCTCTTCGAAACTG
Isg15	GGTGTCCGTGACTAACTCCAT	TGGAAAGGGTAAGACCGTCCT
Zbp1	AAGAGTCCCCTGCGATTATTT	TCTGGATGGCGTTTGATTGG
Mlkl	AATTGTACTCTGGGAAATTGCCA	TCTCCAAGATTCCGTCCACAG
Tnf	AGCCAGGAGGGAGAACAGAAAC	CCAGTGAGTGAAAGGGACAGAACC
Casp3	ATGGAGAACAACAAAACCTCAGT	TTGCTCCCATGTATGGTCTTTAC
Casp8	TGCTTGGACTACATCCCACAC	TGCAGTCTAGGAAGTTGACCA
Ripk3	TCTGTCAAGTTATGGCCTACTGG	GGAACACGACTCCGAACCC
